# Eco-innovation influence on business performance in Jordanian micro, small and medium enterprises operating in the food processing sector

**DOI:** 10.1371/journal.pone.0281664

**Published:** 2023-02-15

**Authors:** Reham Al-Hanakta, Md Billal Hossain, László Pataki, Anna Dunay

**Affiliations:** 1 Doctoral School of Economic and Regional Sciences, Hungarian University of Agriculture and Life Sciences, Gödöllő, Hungary; 2 Hungarian National Bank—Research Center, John von Neumann University, Budapest, Hungary; Rzeszow University of Technology: Politechnika Rzeszowska im Ignacego Lukasiewicza, POLAND

## Abstract

Innovative performance is a fundamental asset for building competitive advantage of micro, small and medium enterprises MSMEs. This research empirically examines the direct and indirect relationship between eco-innovation and business performance in Jordanian MSMEs enterprises working in the food processing sector. This research draws on the resource-based view theory to investigate the inter-relationships among three types of eco-innovation (process, product, organizational) and their relative impact on business performance. Furthermore, the researchers used structural equation modelling of 86 samples collected from Jordanian MSMEs operating in the food processing sector. The major contribution of this research is providing a holistic view that explains the inter-relationship among eco-process, eco-product, and eco-organizational innovation. The research reveals the impact of eco-innovation variables on business performance. The greatest is the impact of eco-process on business performance followed by eco-product and eco-organizational respectively. Regarding the effect of eco-organizational and eco-process innovation on eco-product, the findings of the study showed that the greatest is the effect of eco-organizational followed by eco-process. According to the post hoc, the mean differences show that there is statically significant difference in the responses of the respondents towards eco- process regarding different organization age.

## Introduction

According to its definition, eco-innovation is the production, assimilation or exploitation of a product, production process, service, or management or business methods that are novel to the organization (developing or adopting them) and which results, throughout its life cycle, in a reduction of environmental risk, pollution, and other adverse impacts of resources used (incl. due to pressure from the government and the market, developing a successful eco-innovation program and making it a crucial component of manufacturing sustainability is becoming more and more crucial.

The Organization for Economic Co-operation and Development (OECD) highlighted the two characteristics that set eco-innovation apart from innovation to clarify what it means. The first benefit is that it is an innovation that reflects the concept’s explicit emphasis on a reduction of environmental impact, whether such an effect is intendant or not. Additionally, it includes innovation in social and institutional structures as well as innovation in products, processes, and organizational methods, according to the second tenet [1].

In practice, there are various types of eco-innovation [2], including product innovations, process innovations, organizational innovations, and marketing innovations. While each type of innovation has its own attributes, determinants, and contribution to environmental performance [3], researchers have cautioned that it is not effective to implement innovation programs separately without a systemic view [3, 4]. Nonetheless, previous studies have mostly focused on the development and performance of individual eco-innovation programs, such as product service innovation, service innovation, technological innovation and infrastructure and policy innovation [5].

Besides the recommendations of previous scientific works [6], researchers have addressed eco-innovation from the following perceptions: first, the studies that identify factors driving eco-innovation and the performance outcomes arising from eco-innovation. Second, the studies identifying the dimensions of eco-innovation. The third research category focus on developing an instrument to measures innovation.

Environmental performance measures how well businesses interact with their surroundings, including how they use and manage natural resources and how they manage pollution. The International Organization for Standardization’s Technical Committee, also known as (ISO 14000), has created international standards for environmental management and environmental performance measurement. These policies also had a global component. Multiple Environmental Agreements (MEAS) are a collection of 200 international agreements that deal with environmental issues; on the other hand, the World Bank policy established a number of guiding principles that must be followed when funding development projects by the World Bank, which collectively reflects the absence of financing for environmentally harmful projects [7].

Environmental performance, as before, is the effectiveness of environmental management in reducing pollution and protecting the environment by concentrating on sectorial environmental policies that focus on the production side using tools to measure and evaluate environmental performance through environmentally sustainable performance assessment, product life cycle assessment, and environmental auditing to determine the level of compliance. The facility for environmental laws, as well as the performance measurement model to choose the most efficient mode of production [8. 9].

It might be ineffective to develop eco-innovation without taking a comprehensive approach. For instance, a number of researchers focused solely on technology when addressing eco-innovation issues [7]; According to the socio-technical system theory, implementing innovations should be done in conjunction with appropriate social and managerial systems to maximize business performance, additionally, a company must be able to tweak and refine its internal operations and structure to support technological aspects of eco-innovation [10]. Additionally pointing out that the R&D unit should not be solely responsible for an effective eco-innovation program [11]. An organization must instead develop and support its eco-innovation programs in a comprehensive manner.

Eco-organization implementation refers to organizational members’ competences and commitment to implement new forms of eco-innovation management. Eco-organizations cannot decrease environmental impact directly, but they can assist the implementation of eco-processes (e.g., in manufacturing) and eco-product innovations [12].

The implementation of eco-innovation in eco-organizations includes co-training programs, eco-product design programs, the introduction of eco-learning techniques, the creation of management teams to deal with eco-issues, and eco-management systems [13–15].

The primary theoretical framework for investing in eco-innovation presupposes that stakeholders and regulatory bodies are under pressure and that eco-innovation does not result from the company’s mission in terms of its business practices [16, 17]. This strategy can be contrasted with innovation theory, which views innovation as pressure the company applies to the market rather than the other way around [18].

The food processing sector directly affects human health in terms of nutrition and in terms of food hazards. Medium and small companies can only advance well and become sustainable if their human resources are well trained and educated on food safety measures and laboratory testing. Although this sector is highly regulated and regularly monitored by the Jordan Food and Drug Administration (JFDA), it is composed of many informal small businesses–home businesses, farm businesses or small shops selling dairy products, Arabic sweets, jams, pickles, etc.–mostly distributed around Amman and in the governorates. There is a definite tendency to hire more women in certain activities requiring tolerance and intensive manual work, as well in quality control activities.

The food processing, agriculture and animal husbandry sectors currently employ 52,143 people and generate 4.10 billion Jordanian dinars in income (output). These sectors represented 6.3% of GDP as per figures from 2015, while in 2016; the sector’s exports reached 524.8 million Jordanian dinars representing 10.2% of Jordan’s total industrial exports. According to recent statistics from 2021 (data gathered from the General Department of Statistics for the year 2021), these sectors account for 6.2% of the GDP. 541.1 million dinars worth of exports from this industry made up 10.1% of all industrial exports from Jordan.

The current relevance of the sector is attributed to the fact that it is highly diversified, including all sizes of businesses. More than 95% are MSMEs, 80% of which are micro and small enterprises. Also, this relevance is derived from the forward and backward linkages of the sector in the economy, its degree of integration and the added value generated as a result of these linkages. The sector represents 25.9% of the net added value within Jordan’s industrial economy. Therefore, the sector has become a strategic one both in industry and agriculture [19].

By examining the connections between various forms of environmental innovation and their effects on business performance, this study aims to provide a comprehensive view of environmental innovation programs in response to the literature’s call for such a study. The discussion that followed was specifically focused on the research methodology, including samples and measurements. Following the presentation and discussion of the statistical findings, management implications and recommendations for future research are made.

## Literature review

### Eco-innovation

The literature separates external and internal eco-innovation at the eco-innovation boundary. The organization’s external activities for green and sustainable activities, such as suppliers, regulators, and market demand, are all included in the external boundary of eco-innovation [20]. The practices for effectively and efficiently managing eco-innovation processes within organizations, such as organizational management [21], production process [7] and new product development, are related to the internal boundary of eco-innovation activities [22]. In this study, we concentrate on the internal limit of eco-innovation.

Researchers have also examined eco-innovation from a variety of angles, such as those related to government policy [23], stakeholders (such as clients and suppliers) [24], organizational strategies [25], organizational leadership, organizational culture, and the characteristics of the eco-innovation itself [26]. This study examines the outcomes of eco-innovation from the organizational strategic perspective, with a focus on the internal boundary of eco-innovation.

An extensive literature review was conducted to ensure inclusion of all relevant aspects of the internal boundary of eco-innovation. Eco-innovation types are classified into process/product innovation, mature/immature innovation, and radical/incremental innovation [27]. Other researchers [28, 29] studied three types of eco-innovation: eco-process, eco-product, and eco-organizational innovations. The Oslo Manual, developed by the OECD [2], identified four distinct types of eco-innovation: product innovation, process innovation, organizational innovation, and marketing innovation. Overall, for examining internal innovation, the literature seems to suggest a focus on eco-process, eco-product, and eco-organizational innovation activities [28, 29].

An innovation in an organization’s eco-process is a new component added to its eco-product production system [30]. Eco-process innovation, in general, refers to the enhancement of current production processes or the addition of new processes to lessen the impact on the environment. According to [31] innovation can take the form of additive solutions (such as smokestack scrubbers) or it can be incorporated into the production processes through input substitution, production optimization, and output reclamation. Eco-process innovation therefore alters an organization’s operational procedures and systems, lowers production unit costs, generates new or significantly enhanced eco-products, and lessens environmental impacts [30].

Given the growth that small and medium-sized businesses have seen, green innovation or eco-innovation is therefore seen as a foundation for fostering and growing them. As a result of the emergence of what is known as the "environmental responsibility," which falls on these institutions if they do not take the environmental performance into consideration, it became necessary for its commitment to achieving environmental performance after it aimed to achieve performance or economic effectiveness. The paradigm developed by calls for dynamic capacities from a resource-based perspective (RBV) for organizational, process, and product eco-innovation, which may have an impact on corporate performance [32].

Environmental innovation and performance have a positive impact on costs, sales of distinctive products, profit margins, brand value, and the company’s standing in the community in addition to lowering environmental risks. Designs that take into account economic, social, and environmental factors are among the advancements in environmental regulation [33]. However, big businesses frequently spend a lot of money on R&D, which makes it simpler to adopt changes to environmental laws. On the other side, by recycling and reusing materials, micro and small firms can apply innovation in their operations and products. Recycling of materials is a process that demonstrates environmental innovation.

Therefore, eco-advancements in manufacturing that recycle and reuse raw resources reduce production costs. The creation of an environmental innovation process requires the inclusion of elements that take into account human health, reduce environmental impacts, and abide by laws set forth by governmental bodies. The product’s life cycle is the focus of the environmental advances. By increasing productivity and enhancing the efficiency of production processes, they are able to lessen the environmental impact by using less material and energy [32].

Because of this, decision-makers need to understand that adopting environmental innovation is now the only way for businesses to develop and expand. Many local and foreign clients and buyers require that their suppliers make items that do not include harmful and poisonous substances as environmental innovation have become more crucial for businesses to enhance environmental awareness. Businesses are likewise looking for ways to produce goods with less energy and material input [34].

Last but not least, an eco-organizational innovation is the upgrading of the organization’s management processes through new and eco method in business practices [35]. Thus, eco-organizational innovations can enhance business performance by facilitating necessary adjustments, lowering administrative and transaction costs, enhancing workplace satisfaction [36], or lowering supply-chain costs [37]. Environmental impacts are typically not directly reduced by eco-organizational innovation, but it does make it easier to implement eco-process and eco-product innovations [35]. Positive attitudes towards innovations may facilitate practical implementation of new ideas and processes, which allows the business to achieve sustainable competitive advantage [38].

Eco-organizational innovations, according include eco-training programs, eco-product design programs, eco-learning techniques, or the formation of management teams to address environmental issues. Therefore, administrative efforts to update organizational practices, procedures, mechanisms, or systems to ultimately produce eco-innovations are related to eco-organizational innovations.

### Business performance

There is not a comprehensive body of knowledge in the field of business performance measurement (BP) [39]. Researchers in the field of performance measurement come from a variety of management disciplines, including strategy management, operations management, human resources, organizational behaviour, information systems, marketing, and management accounting and control [39, 40]. Diverse and multidisciplinary research is interesting, but it can also lead to issues. There are numerous definitions of a BPM system as a result of the various approaches to performance measurement, and there is little agreement on its essential features.

Eco-organizational innovation has a direct and favourable impact on economic performance in the Malaysian technology industry, according to empirical research that covered 109 local and foreign-owned technology companies. Furthermore, despite the moderating effect of market turbulence in the Malaysian technology sector, the effects of eco-product and eco-process innovations on environmental performance have been confirmed [41].

According to a study, each of the three eco-innovation categories–eco-process, eco-product, and eco-organization–has a direct bearing on the various aspects of sustainable business performance. Absent the moderation of market turbulence in Ghanaian manufacturing firms, the empirical research findings support the positive effects of eco-product, eco-process, and eco-organization on environmental performance and the positive impact of eco-organization on social performance [42].

According to a study that used data from 442 Chinese companies, eco-innovation behaviour can significantly improve a company’s environmental performance. The study’s findings show that certain factors, such as technological capabilities, environmental organizational capabilities, a market-based instrument, competitive pressures, and customer green demand, contribute to the growth of eco-innovation [43].

Three hundred and sixty-six businesses in the manufacturing and service sectors were chosen as the research sample in order to examine the impact of four types of corporate culture (clan, adhocracy, hierarchy, and market) on the three dimensions of a firm’s environmental innovation (eco-organizational, eco-process, and eco-product). The findings demonstrate that market and adhocracy cultures are positively related to a firm’s eco-organizational, eco-process, and eco-product innovation; clan culture has a positive impact on eco-organizational innovation while hierarchy culture has a negative impact; and the three environmental innovation dimensions all enhance a firm’s financial performance [44].

Cooperation in research and development can have a positive impact on technological eco-innovations of products and processes; cooperation enables eco-innovations to meet environmental requirements and promotes the spread of technical knowledge. 221 electrical and electronic manufacturers with operations in Brazil provided data for a study [45].

R&D capitalization shows a positive correlation with market value. In case of Korean biotech firms, capitalized R&D has a higher value relevance compared to other industries. A large amount of R&D invested in the biotech industry and its capitalization are considered as key to the future sustainable success of these firms [46].

Whether they are focused on domestic or international markets, many organizations around the world are impacted by a variety of factors, which are reflected in their levels of performance and, consequently, their level of success. Here, we will briefly discuss a few of these elements that affect the majority of businesses.

From external factors, the first to be discussed is the economic situation and the extent of its stability, whether at the national or global level. They make it challenging for many establishments to perform at their peak levels and to the fullest extent in the event that there are changes in the economy, whether local or international, in a negative way, as the existence of economic crises is typically reflected on the performance of the markets and causes in various ways. Second main factor is the group of competitors. This is an external hazard that cannot be eliminated in any manner is the growth in the number of rival businesses for the institution. As a result, many businesses are forced to spend more money to keep skilled employees on staff and stop them from defecting to other companies. It is important to note that high-performance practices like flexible rewards and positive manager-employee relationships can help to lessen or even eliminate this kind of threat [33].

From internal factors, the first and most crucial factor is the administrative method. If old administrative method does not help to compete with other modern organizations, the modern horizontal management method is one of the best solutions to raise performance, where all levels share responsibilities within their functional and specific roles, while giving it the necessary powers in order to achieve those roles.

Performance on an individual and team level is another internal aspect that could have a detrimental impact on the establishment’s ability to compete. Everyone has certain demands that the management must be aware of and considerate of, such as fair compensation for the nature and complexity of the job, training, rewards, and other perks that will motivate workers to perform their jobs well and more effectively. In terms of teamwork, the effectiveness of the team is always influenced by the number and quality of its members. For example, selecting the incorrect number of team members to complete a task may increase conflict or cause discord, which has a negative impact on the outcomes. It is crucial that the team’s members are diverse and that the right selection criteria are in place.

If work policies and procedures are not reviewed and updated, it may affect the organization’s performance as a whole, as well as the efforts of the work teams and the achievement of the desired results. It is essential to assess the regulatory standards and update those policies and procedures permanently because they must be in line with the establishment’s plan [34].

### Eco-innovation and business performance

There is no sufficient research in the developing countries focusing on eco-innovation related to sustainability performance of business practices.

The resource-based view (RBV) declares that the maintaining of firms’ competitive advantage lies in having heterogeneous resources that are valued, uncommon, incomparable, and no substitutable. RBV provides a valid theoretical basis for inspecting the relationship between resources, capabilities, and performance. This theory provides an inclusive view of eco-innovation [47]. Business performance is related to accomplishing certain outcomes by transforming the inputs into outputs. The organization’s main goal is to increase their performance to meet the competitive market. There is a variance measurement developed by scholars to measure the organization performance like ROI, market share, profitability, and sales. In this research, quality management was added to performance measurement.

As market share, sales volume, and profit growth signify high growth linked with a firm’s entrepreneurial orientation, entrepreneurial orientation (EO) is a significant component of organizations’ competitive advantage, growth, and performance. Corporate performance is therefore consistent with the characteristics of an entrepreneurial orientation—innovation, risk-taking, and reactiveness. In the body of research on learning orientation, academics have emphasized the significance of an entrepreneurial orientation because it is strategically compatible with company performance and because globalization has increased corporate competition, which has resulted in an expansion of entrepreneurship in many fields. Many learning direction studies have concentrated on the critical role of best management practices as well as the role of entrepreneurial practices resulting in improved corporate performance [48].

Increased competition among businesses and organizations around the world is another manifestation of globalization’s effects. Companies prioritize optimal management practices in their quest for increased performance and productivity. Previous studies have demonstrated the significance of an entrepreneurial orientation for the success of top companies. Additionally, academics have emphasized the critical role that learning direction plays in advancing high-level generative learning, which is a crucial element of unparalleled corporate efficiency and, as a result, elevated corporate performance [49].

Entrepreneurial orientation (EO) is the behavioural propensity for innovation, reactiveness, and risk-taking inside a company that improves organizational performance. EO refers to internal organizational management methods that are innovative and proactive in order to achieve improved performance and acquire a competitive advantage in the market, particularly in small and medium-sized businesses. The internal environment in which a corporation operates can affect corporate performance. These opinions suggest that because there are numerous EO characteristics that represent business performance, EO behaviour cannot be generalized across industries [50].

The readiness of a company to innovate its business operational processes is referred to as innovation. It is an organizational strategy that describes the use of fresh concepts that result in innovative products and services. A corporation can benefit from new prospects through innovation, satisfy consumer requirements with novel products and services, and pioneer a market. Innovation also has to do with the fundamental business procedures that set businesses apart and prolong their survival. Companies improve their standing in the market, which promotes business growth and performance, as a result of innovation and value addition in products and services. Additionally, it is an organizational strategy that mentions the need to take initiative in order to advance [50].

Eco-innovation is a managerial mainstream, which still need more research and investigation. At the same time, it is increasingly becoming a concern of decision makers, academics, and practitioners. Recent studies [51–53] support the notion that process innovation often arms existing production processes with advanced techniques, which, in turn, improves the capability of adding new product features to meet the market’s needs. In a nutshell, the improvement of eco-process innovations is driving force for eco-product innovations [51].

### Research model

**Fig 1:**
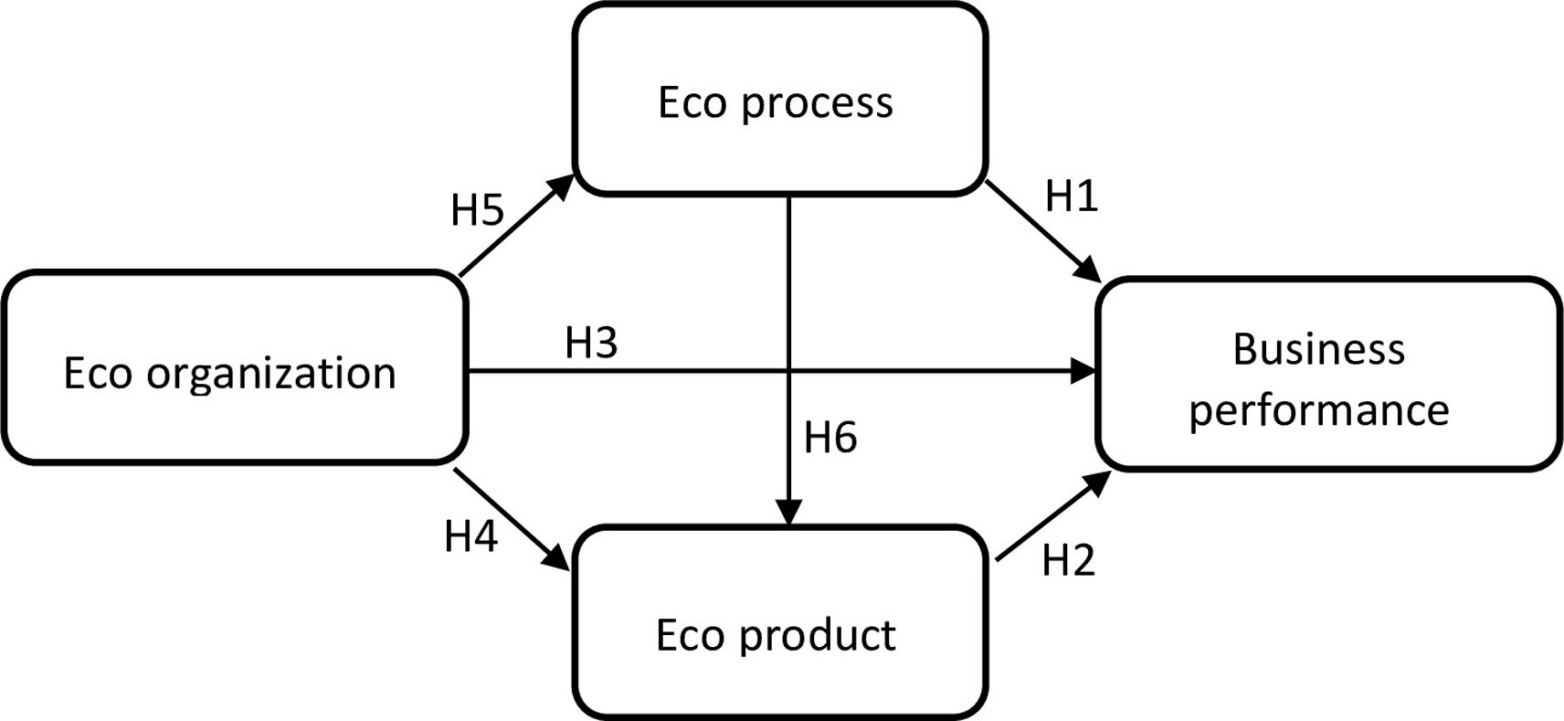
The basic model that displays the theoretical relationship among three types of eco-innovation and business performance.

The above explanation and discussion were made the basis of the development of the proposed research model in Fig 1.

## Materials and methods

### Sample and data collection

The samples were collected from Jordanian companies working in food processing sector outlining the following 11 sub-sectors and hundreds of products: 1) Processed and preserved meat; 2) Dairy products; 3) Processed and preserved fruits and vegetables; 4) Bakery and Arabic sweets; 5) Animal and vegetable fats and oils; 6) Products from the milling industry; 7) Cocoa, chocolate and sugar confectionery; 8) Processed fish and crustaceans; 9) Macaroni and pasta products; 10) Other food products; 11) Animal feed.

The data were analyzed anonymously. The study was reviewed and approved by an institutional review board (ethics committee) of the Doctoral School Hungarian University of Agriculture and Life Sciences before the study commenced.

Average annual growth (turnover) for the main subsectors was around 4.9% between 2013 and 2017. This is mainly attributed to the increase in households and the Syrian refugee population. In terms of market access, certain Jordanian products are gaining ground on the domestic level and in many cases are fully replacing imports, for example in dairy products, Arabic sweets and some processed fruits and vegetables such as dried dates. In terms of food exports, certain indicators display that Jordan has not yet exhausted its market potential, and some sub-sectors are growing at a regular pace and reaching new Western markets, such as Arabic sweets and processed fruit and vegetable sub-sectors, and in ready-to-eat food.

The drastic loss of market share in close markets such as Iraq and Syria is due to regional political instability and is being compensated by other Arab markets, such as the Gulf Cooperation Council (GCC) countries, in many priorities sub-sectors and in the processed meat subsector.

Nevertheless, the regional Arab market remains Jordan’s main export market, accounting for over 75% of its processed food exports. The remaining 25% is mainly shared between North America and Europe [19].

The findings of recent study [19] showed that the main cost factors negatively affecting the competitiveness of all the food sub-sectors were raw materials; cost of utilities such as energy and water; and economies of scale. Raw materials were often imported, which had a rather high negative influence, while scarcity of utilities was highly negatively influential. On the other hand, negative influence of economies of scale was relatively small compared to regional counterparts.

It was also found that small and informal enterprises operating in the subsectors also face other challenges at the production level, such as lack of skilled workforces, dependence on traditional methods, lack of international certification, low involvement of women, and lack of professional hygiene standards. Some challenges (e.g. lack of skilled workers, certifications) might be solved by attracting intellectual migrants to strengthen host countries [54].

### Measurements

The survey method is used in this research to provide an outline of the existing eco-innovation practices and effectiveness in Jordan. 86 questionnaires were sent to MSMEs in the food processing sector, which represent approximately 20 percent of the sector. (The total number of MSMEs in the food processing, agricultural, and animal husbandry sectors is about 400 firms).

Structural equation modelling (SEM) analysis was applied to test the research hypotheses. Online questionnaires were sent to the manufacturing firms that were sampled. Each firm’s senior manager answered all the items on the questionnaire. The research objectives were summarized in the cover letter that accompanied the questionnaire. It was recommended that the general manager respond to inquiries directly or choose a qualified senior manager or high-level assistant on their behalf. Data gathering was conducted in December 2021 and January, February 2022.

The questionnaire included three sections: (1) the covering page with contact information, (2) a series of items on eco-process innovation, eco-product innovation, eco-organizational innovation, and business performance, and (3) demographics (firm size and age).

Eco-innovation items list used in this research was generated. The items were generated including four items measuring the eco-process construct, seven items measuring the eco-product construct, and six items measuring the eco-organizational construct. Business performance was measured using five items, four items of which were developed by Im & Workman [55]. All the items were self-reported by asking respondents to rate their market and financial performance. The ROI, sales, profit, market share and quality were added in this research compared with that of their major competitors. The items were measured on a 5-point Likert scale (1 = strongly disagree; 5 = strongly agree). Based on the mean values of Likert scale points a rank was created, by which the importance of the different items were determined according to the answers of the respondents.

### Data analysis

The results were obtained from 86 respondents. The data were gathered using a questionnaire, and Statistical Package for Social Sciences (SPSS) version 26 is utilized to analyse data.

### Validity

The confirmatory factor analysis (CFA) is a statistical mechanism that was conducted to reduce factors or study items to a smaller set of factors. Based on CFA results, one can decide which items are appropriate and can be used for further analysis, and which one caused cross loading and should be excluded.

In refining the model, the rationality and consistency of the data with theoretical underpinnings in the relationship between eco-innovation and business performance were considered.

The base limits of indices utilized as a part of measuring the Goodness of fit index (GFI) of the measurement model are stipulated [32, 56, 57]. As p value less than 0.05, comparative fit index (CFI) ≥ 0.90, GFI ≥ 0.90, χ2/df (χ2/df) <5 and root mean square error of approximation (RMSEA) ≤0.05–0.80.

The statistics discovered a p-value of 0.000, CFI value of 0.926, GFI 0.903, χ2/df value of 3.635 and RMSEA value of 0.070. This indicated that the fit statistics are adequate and within the acceptable thresholds to establish the convergence validity of the measurement model for the relationship between eco-innovation and business performance.

### Reliability

Cronbach’s’ Alpha Coefficient is a measure that was used to estimate the reliability. Cronbachs’ Alpha Coefficient value ranges between 0 and 1. If there are no variance among study instruments (i.e., are internally independent) then α = 0 but if all study items have a high covariance, then α will be close to 1. However, there is a consistence among researchers that the instrument is considers reliable and stable if α values are more than 0.7.

Table 1 shows that Cronbach’s’ Alpha Coefficient values are more than 0.7, then the instruments of questionnaire are reliable and consistent.

**Table 1:** Reliability statistics.

	Cronbach’s Alpha	N of Items
**Eco-process**	0.873	4
**Eco-product**	0.941	6
**Eco-organization**	0.909	7
**Eco-innovation**	0.913	17
**Business performance**	0.915	5

## Analysis and results

### Demographic profile

Table 2 shows that out of 86 respondents, 28 are working in organization with 5 to 25 employees, forming (32.6%) of the total respondents. Also, 14 respondents are working in organization with 25 to 50 employees, which makes (14%), while 10 are working in organization have 50 to 100 employees representing (11.6%), and 34 are working in organization have 100 and more employees forming (39.5%) of the total respondents.

**Table 2:** Total number of employees.

		Frequency	Percent	Valid Percent	Cumulative Percent
Valid	5–25	28	32.6	32.6	32.6
	25–50	14	16.3	16.3	48.8
	50–100	10	11.6	11.6	60.5
	= > 100	34	39.5	39.5	100.0
	Total	86	100.0	100.0	

Table 3 illustrates that out of 86 respondents, 11 are working in organization aged between 3 to 5 years representing (12.8%), 11 are working in organization aged between 5 to 10 years forming (12.8%), while 40 are working in organization aged between 10 to 25 years, which represents (46.5%), 5 are working in organization aged between 25 to 50 years with a percentage of (5.8%), and 19 are working in organization aged between 50 to 100 years representing (22.1%).

**Table 3:** Age of company.

		Frequency	Percent	Valid Percent	Cumulative Percent
Valid	3–5	11	12.8	12.8	12.8
	5–10	11	12.8	12.8	25.6
	10–25	40	46.5	46.5	72.1
	25–50	5	5.8	5.8	77.9
	50–100	19	22.1	22.1	100.0
	Total	86	100.0	100.0	

Table 4 shows that out of 86 respondents, 1 is working in agricultural organization representing (1.2%), 2 are working on business support organization, which represents (2.3%) and 83 are working in food manufacturing organization forming a percentage of (96.5%).

**Table 4:** Economic activity.

		Frequency	Percent	Valid Percent	Cumulative Percent
Valid	Agriculture	1	1.2	1.2	1.2
	Business support organization	2	2.3	2.3	3.5
	Manufacturing	83	96.5	96.5	96.5
	Total	86	100.0	100.0	

### Descriptive and ranking analysis

This section presents the descriptive analysis of the data obtained from the respondents regarding the study’s questions. The section aims to show the perceptions of the respondents regarding eco-innovation and business performance. For this purpose, the mean and standard deviation values of Likert points were used.

Eco-innovation part of the questionnaire was to assess and rank eco-innovation dimension. The respondents were asked to evaluate a set of statements related to 3 groups: eco-process, eco-product and eco-organization. Respondents were asked to rate their firms on a 5-point Likert scale according to the statements presented in Table 5. The mean values were calculated from the Likert scale points, and the rank was determined based on the mean values.

**Table 5:** Descriptive statistics (ECO innovation).

Statements in the questionnaire	Mean	Std. Deviation	Rank
Our firm often innovatively updates manufacturing processes to protect against contaminations	3.86	1.08	4
Our firm often innovatively updates manufacturing processes to meet standards of environmental law	3.93	0.78	2
Our firm often uses innovative technologies in manufacturing processes to save energy	4.07	0.79	1
Our firm often innovatively updates manufacturing equipment in manufacturing processes to save energy	3.90	0.95	3
**ECO Process**	**3.94**	**0.71**	**1**
Our firm often places emphasis on developing new eco-products through new technologies to simplify their package	3.62	0.90	6
Our firm often places emphasis on developing new eco-products through new technologies to simplify their construction	3.67	0.86	3
Our firm often places emphasis on developing new eco-products through new technologies to easily recycle their components	3.65	0.88	4
Our firm often places emphasis on developing new eco-products through new technologies to easily decompose their materials	3.63	0.90	5
Our firm often places emphasis on developing new eco-products through new technologies to use natural materials	3.71	1.03	2
Our firm often places emphasis on developing new eco-products through new technologies to reduce damage from waste as much as possible	3.85	0.86	1
**ECO Product**	**3.69**	**0.76**	**2**
Our firm often places emphasis on developing new eco-products through new technologies to uses little energy as possible	3.93	0.89	1
Our firm’s management often uses novel management systems to manage eco-innovation	3.45	0.82	5
Our firm’s management often collects information on eco-innovation trends	3.45	0.88	6
Our firm’s management often actively engages in eco-innovation activities	3.58	0.93	2
Our firm’s management often communicates eco-innovation information with employees	3.55	0.90	3
Our firm’s management often invests a high ratio of R and D in eco-innovation	3.31	0.96	7
Our firm’s management often communicates experiences among various departments involved in eco-innovation	3.50	0.76	4
**ECO Organization**	**3.54**	**0.67**	**3**
**ECO Innovation**	**3.72**	**0.61**	

As presented in Table 5, the highest ranked group as a highest important group is “ECO Process” where the least ranked group is “ECO Organization”.

It is seen from Table 5 that ECO Innovation has a mean of 3.72 and a standard deviation of 0.61. Eco Innovation consists of three parts: ECO process, ECO product, and ECO Organization.

ECO process was ranked one as the most important factor has a mean of (3.94) and standard deviation of (0.71). On the one hand, a consensus exists when it comes to paying attention to innovatively updating manufacturing process to meet environmental law standards with a mean of (3.93), and on using innovative, energy saving technologies with a mean of (4.07) and they got a rank of two and one respectively. On the other hand, much lesser attention is paid to when it comes to keeping manufacturing processes innovatively up to date against contaminations that unexpectedly got a rank of four with a mean of (3.86), and rank three when it comes to keeping the manufacturing equipment innovatively updated in order to save energy with a mean of (3.90). This implies that firms do not make effort to keep their manufacturing processes up to date to protecting themselves from contaminations, and to save energy.

ECO product has a Mean of (3.69) and Standard deviation of (0.76). It can be concluded from the table that firm’s focal point relies on developing new eco-products to simplify their construction with a mean of (3.67), to use natural materials with a mean of (3.71), or to reduce damage from waste with a mean of (3.76), that got ranked three, two, and one respectively. However, firms give much lesser focus when it comes to developing new eco-products using new technologies to simplify packing, to easily decompose their materials, and to easily recycle their components, that got a rank of six, five, and four respectively.

ECO Organization was ranked as the least important factor of ECO innovation with a mean of (3.54) and standard deviation (0.67). It is seen in Table 5 that firm’s management place emphasis on developing new ECO-products through technologies that uses as much little energy as possible with a mean of (3.93) that was ranked one in this section and engaging in Eco-innovative activities comes in second rank with a mean of (3.58). Nonetheless, unexpectedly, spending on eco-innovative research and development is the last thing on firms’ management To-Do list with a mean of 3.31 and got the least rank of 7. Likewise, collecting information on ECO-innovation trends was ranked 6 with a mean of 0.88.

Business Performance part of the questionnaire was to assess and rank business performance items, the respondents were asked to evaluate a set of items related to their business performance during the last three years. Respondents were asked to rate their firms on a 5-point Likert scale according to the items presented in Table 6. The mean values were calculated from the Likert scale points, and the rank was determined based on the mean values.

**Table 6:** Descriptive statistics (Business performance).

**Items**	**Mean**	**Std. Deviation**	**Rank**
Return on investment	3.49	0.79	5
Profits	3.57	0.70	2
Market Share	3.56	0.78	4
Sales	3.56	0.71	3
Quality Management	3.78	0.71	1
**Business performance**	**3.59**	**0.60**	

As displayed in Table 6, the highest ranked item as most important factor of business performance was “quality management”, second was “profit”. “Market share” and “sales” were almost similar, while the least ranked item according to the respondents was “return on investment”. Quality has a higher importance in the food sector, which is reflected by these results.

Jordanian food standards are recognized either internationally or at the regional level, according to the type of food product. The Jordan Quality Mark (JQM)is granted for food industries based on product compliance with technical specifications and the system’s agreement with the International Organization for Standardization (ISO) 9001:2000 and ISO 22000:2005.There are also private sector certifying bodies, such as Lloyd’s Register, Occupational Health and Safety (OHAS), ISO 14000, Hazard Analysis of Critical Control Points (HACCP), British Retail Consortium Global Standard for Food Safety (BRCGS), Food Safety System Certification (FSSC) 22000, in addition to several other certifications.

### Hypothesis testing

This part of the study aims to examine the impact of eco-innovation on business performance by testing the following Hypotheses:

**H1:** The greater the firm’s eco-organizational innovation, the greater its eco-process innovation.

**H2:** The greater the firm’s eco-organizational innovation, the greater its eco-product innovation.

Although previous general innovation studies have provided theoretical support for the relationship between eco-process innovation and eco-product innovation, the literature does not explicitly provide empirical results for this relationship. For better results, product innovation must come before process innovation. On the other hand, product and process innovations are complementary to one another and that businesses that pursue both simultaneously would perform better [58].

Last but not least, a variety of process innovation activities, including setting up new machinery, redefining task specifications, and improving information flow could aid in the development of new products [59, 60]. In order to encourage product innovation, a functionally new manufacturing process (such as a reduction in production unit costs) is required [61]. Numerous recent studies [51–53], seem to support the idea that process innovation frequently equips existing production processes with cutting-edge techniques, which, in turn, improves the capability of adding new product features to meet the needs of the market. In other words, eco-product innovations are propelled by the advancement of eco-process innovations. Consequently, the following theory is put forth.

**H3:** The greater the firm’s eco-process innovation, the greater its eco-product innovation.

Successful innovations, according to many studies on the relationship between innovation and performance, enhance business performance. For instance, [62] validated the advantages of product innovation and investigated the effects of process innovation, and [63] investigated the effects of process innovation. The impact of environmental management (including all three types of eco-innovation) on business performance has also been acknowledged [64]. The connections between proactive environmental strategy (including innovation) and business performance as well as the development of organizational capabilities through environmental practices have been amply supported by numerous publications, for example [65–67]. They all backed the idea that process/product innovations and business performance are positively correlated. In light of this, we suggest that company performance.

**H4:** The greater the firm’s eco-process innovation, the greater its business performance.

**H5:** The greater the firm’s eco-product innovation, the greater its business performance.

Previous studies have also argued that eco-organizational innovations, such as innovative design, speed, or flexibility, can improve a firm’s business performance in addition to conventional organizational innovations [66]. According to strategic theories, companies that adopt innovations (such as capabilities, resources, technologies, or knowledge of the innovation) will subsequently develop a special mechanism to safeguard profit margins, allowing the company to benefit greatly [68] while another research [22] specifically linked increased business performance and organizational innovation. Consequently, the following theory is put forth.

**H6:** The greater the firm’s eco-organizational innovation, the greater its business performance.

The study can carefully examine both the direct and indirect effects of innovations in eco-processes and eco-organizations thanks to these research hypotheses. Particularly, innovations in eco-processes, eco-products, and eco-organizations all directly enhance business performance (H4, H5 and H6). Additionally, eco-process innovation (H1) and eco-product innovation (H2) act as mediators between eco-organizational innovation and business performance, and eco-product innovation also mediates the relationship between eco-process innovation and business performance (H3).

The performance of businesses can be indirectly improved through eco-product innovation, eco-process innovation, and eco-organizational innovation. Eco-product innovation enables a business to apply its internal innovative initiatives to creating new products or services. To improve business performance, those activities also support eco-process innovation. Therefore, it is likely that both direct and indirect effects from eco-product innovation will be included in the overall effects of eco-organizational and eco-process innovation on business performance. Previous studies have shown that other types of capabilities, such as those related to operations or marketing, can act as mediators in the capability-performance relationship [69, 70]. As far as we are aware, no studies have looked into how the three types of eco-innovation interact with one another.

Table 7 presents the structural model parameters with the hypothesized relationships and the hypothesis test results. The table illustrates that with at 95% confidence, the *P*-values are 0.000 which is less than α = 0.05. Therefore, the null hypotheses are rejected. In other words, there is a significant impact of eco-process, eco-product and eco-organizational on Business performance as well as a significant impact of eco-organizational on eco-process and eco-product and a significant impact of eco-product on eco-process.

**Table 7:** Hypotheses testing results.

	Estimate	S.E.	C.R.	P-value
Environmental organizational innovation → environmental processes	0.486	0.30	16.109	0.000
Environmental regulatory innovation → environmental product innovation	0.412	0.026	15.902	0.000
Environmental process innovation → environmental product innovation	0.236	0.022	10.539	0.000
environmental processes → performance business	0.536	0.031	17.549	0.000
Environmental product innovation → business performance.	0.758	0.035	20.703	0.000
Environmental regulatory innovation → business performance.	0.596	0.034	17.290	0.000

Regarding the impact of eco-innovation variables on Business performance, the greatest effect is of eco-process on business performance followed by eco-product and eco-organizational respectively. Additionally, regarding the effect of eco-organizational and eco-process on eco-product, the findings show that the greatest effect is of eco-organizational on eco-product followed by the effect of eco-process on eco-product.

Table 8 illustrates the effect of each item on its variable.

**Table 8:** Items results.

	Estimate	S.E.	C.R.	P-value
**ECO Organizational**				
Our firm often places emphasis on developing new eco-products through new technologies to uses little energy as possible	0.750	0.049	21.879	0.000
Our firm’s management often uses novel management systems to manage eco-innovation	0.601	0.048	23.256	0.000
Our firm’s management often collects information on eco-innovation trends	0.912	0.046	22.293	0.000
Our firm’s management often actively engages in eco-innovation activities	0.800	0.048	22.477	0.000
Our firm’s management often communicates eco-innovation information with employees	0.733	0.050	21.344	0.000
Our firm’s management often invests a high ratio of R and D in eco-innovation	0.563	0.053	21.223	0.000
Our firm’s management often communicates experiences among various departments involved in eco-innovation	0.611	0.047	21.668	0.000
**ECO Product**				
Our firm often places emphasis on developing new eco-products through new technologies to simplify their package	0.622	0.042	24.295	0.000
Our firm often places emphasis on developing new eco-products through new technologies to simplify their construction	0.543	0.051	24.428	0.000
Our firm often places emphasis on developing new eco-products through new technologies to easily recycle their components	0.641	0.040	25.603	0.000
Our firm often places emphasis on developing new eco-products through new technologies to easily decompose their materials	0.939	0.043	23.583	0.000
Our firm often places emphasis on developing new eco-products through new technologies to use natural materials	0.471	0.045	24.583	0.000
Our firm often places emphasis on developing new eco-products through new technologies to reduce damage from waste as much as possible	0.891	0.048	23.941	0.000
**ECO Process**				
Our firm often innovatively updates manufacturing processes to protect against contaminations	0.971	0.045	21.342	0.000
Our firm often innovatively updates manufacturing processes to meet standards of environmental law	0.714	0.047	23.367	0.000
Our firm often uses innovative technologies in manufacturing processes to save energy	0.931	0.049	21.901	0.000
Our firm often innovatively updates manufacturing equipment in manufacturing processes to save energy	0.713	0.061	21.119	0.000
**Business Performance**				
Return on investment	0.926	0.062	14.893	0.000
Profits	0.906	0.064	14.177	0.000
Market Share	0.863	0.061	14.243	0.000
Sales	0.971	0.065	14.817	0.000
Quality Management	0.823	0.065	14.581	0.000

### ANOVA test

**H7**: There are insignificant differences of the respondents’ response toward the study’s variables regarding different organization size.

ANOVA at 95% confidence interval was conducted to test this hypothesis. As displayed in Table 9, the p values are more than 0.000. Hence, there is no statically significant difference in the respondents’ responses towards study variables regarding different organization size.

**Table 9:** ANOVA (7^th^ hypothesis).

		Sum of Squares	Df	Mean Square	F	Sig.
ECO Process	Between Groups	1.021	2	.510	1.015	.367
	Within Groups	41.721	83	.503		
	Total	42.742	85			
ECO Product	Between Groups	.813	2	.406	.697	.501
	Within Groups	48.398	83	.583		
	Total	49.211	85			
Eco-organization	Between Groups	.460	2	.230	.508	.604
	Within Groups	37.557	83	.452		
	Total	38.016	85			
Business performance	Between Groups	.320	2	.160	.437	.648
	Within Groups	30.356	83	.366		
	Total	30.676	85			

**H**_**8**_: There are insignificant differences of the respondents’ response toward the study’s variables regarding different organization age.

ANOVA at 95% confidence interval was conducted to test this hypothesis. As presented in Table 10, the p values are less than 0.000 for eco-process. Hence, there is statically significant difference in respondents’ responses towards eco-process regarding different organization age.

**Table 10:** ANOVA (8^th^ hypothesis).

		Sum of Squares	df	Mean Square	F	Sig.
ECO Process	Between Groups	6.918	4	1.729	3.910	.006
	Within Groups	35.824	81	.442		
	Total	42.742	85			
ECO Product	Between Groups	.139	4	.035	.057	.994
	Within Groups	49.072	81	.606		
	Total	49.211	85			
ECO Organization	Between Groups	.212	4	.053	.113	.977
	Within Groups	37.805	81	.467		
	Total	38.016	85			
Business performance	Between Groups	1.290	4	.323	.889	.474
	Within Groups	29.386	81	.363		
	Total	30.676	85			

Most food processing ventures require four or five years before they show a good internal rate of return on investment. There is also an absence of sufficient subsidized loans and grants allocated to R&D; and most of these loans currently come from external donors [19].

Post-hoc test was conducted for the hypothesis that has significant differences in order to find that these differences as shown in Table 11.

**Table 11:** Scheffe test results.

Dependent Variable			Mean Difference (I-J)	Std. Error	Sig.	95% Confidence Interval	
						Lower Bound	Upper Bound
ECO Process	3–5	5–10	-.22727	.28357	.958	-1.1212	.6667
		10–25	-.75511*	.22641	.032	-1.4689	-.0414
		25–50	-.48636	.35869	.765	-1.6171	.6444
		50–100	-.75478	.25196	.071	-1.5491	.0395
	5–10	3–5	.22727	.28357	.958	-.6667	1.1212
		10–25	-.52784	.22641	.256	-1.2416	.1859
		25–50	-.25909	.35869	.971	-1.3898	.8717
		50–100	-.52751	.25196	.364	-1.3218	.2668
	10–25	3–5	.75511*	.22641	.032	.0414	1.4689
		5–10	.52784	.22641	.256	-.1859	1.2416
		25–50	.26875	.31545	.947	-.7257	1.2632
		50–100	.00033	.18530	1.000	-.5838	.5845
	25–50	3–5	.48636	.35869	.765	-.6444	1.6171
		5–10	.25909	.35869	.971	-.8717	1.3898
		10–25	-.26875	.31545	.947	-1.2632	.7257
		50–100	-.26842	.33426	.957	-1.3222	.7853
	50–100	3–5	.75478	.25196	.071	-.0395	1.5491
		5–10	.52751	.25196	.364	-.2668	1.3218
		10–25	-.00033	.18530	1.000	-.5845	.5838
		25–50	.26842	.33426	.957	-.7853	1.3222

## Conclusion

We developed a research model that illustrates the relative significance of each type of eco-innovation and the nature of their interdependency, drawing on the existing innovation literature and the field studies. There has never been a clear consensus regarding how eco-organizational innovation can influence the development of eco-product or eco-process innovations, despite the possibility of a relationship between organizational innovation and product/process innovation suggested by earlier studies. Our statistical findings imply that eco-organizational innovation partially mediates the effects of eco-process and eco-product innovations, and that eco-process innovations partially mediate the effects of eco-product innovations on business performance. Eco-organizational, eco-process, and eco-product innovations have a direct and indirect impact on business performance.

Several research contributions are noteworthy. First, the importance of systematically implementing various aspects (technological, sustainable, social, organizational, etc.) of innovation programs has been previously suggested. However, the current eco-innovation research does not offer a holistic view of eco-innovation [71, 72]. As indicated by [73], to achieve green and sustainable manufacturing requires a holistic view for spanning product, manufacturing processes, and managerial systems across multiple product life cycles. As such, a major contribution of this study to the eco-innovation literature is to employ the RBV theory to frame a conceptual model that links organizational resources (three types of eco-innovation) and business performance, and therefore, provide a holistic view in explaining the inter-relationship among eco-process, eco-product, and eco-organizational innovations. Consequently, a holistic view of the eco-innovation program provides a valuable, inimitable, and non-substitutable resource that should enable a firm to develop the competence leading to better business performance.

Second, the connections between the three eco-innovation components and their effects on business performance imply that each component may be used both singly and collectively. The literature on eco-process innovation, in particular, has frequently concentrated on cost reduction or operating system adjustment, failing to acknowledge the necessity of discussing how process innovation facilitates eco-product innovation or mediates eco-organizational innovation. In addition, our research contributes to the body of knowledge on eco-innovation by illuminating the fundamental role that eco-organizational innovation plays in the development of eco-process and eco-product innovations. Evidently, managers would not be able to implement successful eco-innovation programs without a systemic view of all three types of eco-innovation concurrently.

In order to enhance business performance, management must fully comprehend the relative advantages and restrictions of each eco-innovation type. In contrast to eco-product innovation, eco-organizational and eco-process innovations can directly assist businesses in achieving better business performance. However, because of their effects on eco-product innovation, both eco-organizational and eco-process innovations can achieve better business performance. Therefore, management must rely on, invest in, and implement all three types of eco-innovation when adopting them, with a focus initially on eco-organizational innovation.

The findings of this research could provide policy makers with guidelines for creating environmental regulations that will effectively encourage the creation of eco-innovation programs in business. Resources would be scarce, which would impede the advancement of environmental management. According to the literature, effective environmental regulation could either compel or incentivize business to take environmental measures that improve resource efficiency and environmental productivity [47, 64]. Some measures voluntary implemented by companies have clear evidence of their positive influence on competitiveness, such as utilization of social capital which is a key element of further firm development [74]. However, there is a need for constant environmental, social, and governance (ESG) practices supported both on entrepreneurial and country level [75, 76].

Our findings suggest that a systemic approach is necessary for a successful eco-innovation program. Programs for administrative support must be put in place, in particular, to improve eco-technological innovation’s components. In general, administrative actions do not directly lessen environmental impacts, and managers are more likely to emphasize technological aspects of innovation. Considering the indirect effects of eco-organizational innovation, resources invested in eco-product and eco-process innovations would not be as effective without the required administrative support and procedures. As a result, policymakers should impose rules or provide incentives to encourage businesses to develop and implement eco-innovation programs in a more efficient order.

The findings imply that firms should first engage in eco-process by updating manufacturing processes to save energy, meet standards of environmental law and update process to protect against contaminations. Since process innovation displays the strongest direct effect on business performance, the enhancement of eco-process innovations is a driving force for eco-product innovations.

Eco-organization innovation enables eco-product innovation followed by the effect of eco-process on eco-product, moreover, eco-organizational innovation indirectly affects business performance via the mediators, eco-process innovation and eco-product innovation.

Without having a systemic view of all three types of eco-innovation simultaneously, managers would not be able to apprehend effective eco-innovation programs.

Another interesting finding, according to the post hoc mean differences show that there is statically significant difference in the respondents’ responses towards eco-process regarding different organization age, namely those aged 3–5 years.

The result of this research provides the policy makers with guidelines regarding developing operational environmental regulations to enforce the development of effective eco-innovation programs in the food processing industry.

## Recommendations

Despite the growing significance of the food processing sector for Jordan’s economy, there are no national strategies or policies apparent to stimulate the sector. The most recent initiative addressing the food processing sector is the Jordan Vision 2020 initiative.

Hence, there is need to strengthen the joint and focused support for the food processing sector from the government and the chambers following a developed strategy, given that–unlike other sectors–there is a large prevalence of microenterprises that are not well represented within the chambers, for obvious reasons related to size and registered capital.

The processed foods sector lacks a specific association. Instead, the needs of the sector are mostly addressed through the Chambers of Industry and the SMEs Association. Micro and small food processing enterprises are underrepresented in the Chambers of Industry because of the dominance of medium and large enterprises in the sector.

The means to support innovation can be provided by linking the industry’s needs with the academic resources. The success of applied research projects necessitates both parties to have clear understanding of the required output and a backed source of finance or grants. MSMEs would benefit from being able to depend on public research centers such as National Energy Research Centre (NERC), which assumes the development and research projects and delivers training on new and renewable energy and efficient energy utilization, as well as the National Centre for Agricultural Research and Technology Transfer (NCARTT); public universities; and technology schools.
